# Editorial: Prevention and treatment of skin diseases

**DOI:** 10.3389/fphar.2025.1753830

**Published:** 2026-01-08

**Authors:** R. Di Liddo, F. Caroppo, M. Kawasumi, B. Zavan

**Affiliations:** 1 Department of Pharmaceutical and Pharmacological Sciences, University of Padova, Padova, Italy; 2 Dermatology Unit, Department of Medicine & Department of Women’s and Children’s Health, University of Padova, Padova, Italy; 3 Department of Dermatology, University of Washington School of Medicine, Seattle, WA, United States; 4 Department of Dermatology, The Ohio State University College of Medicine, Columbus, OH, United States; 5 The Ohio State University Comprehensive Cancer Center, Columbus, OH, United States; 6 Department of Translational Medicine, University of Ferrara, Ferrara, Italy

**Keywords:** skin disease, biologics, enzymes, precision medicine, immunotherapy

Over the past decade, significant progress has been made in the prevention and treatment of skin diseases, driven by therapeutic/technological advances, as well as an increased understanding of the pathophysiology of dermatological disorders. In this context, we compiled the scientific contributions of experts in this field to draw readers’ attention to the latest approaches in dermatology, emerging from both clinical and basic research. A total of 14 manuscripts is included in this Research Topic, covering the areas of wound healing, UVB damage, autoimmune/inflammatory diseases, benign skin tumors, and genetic disorders (Table 1).

**TABLE 1:** Scientific contributions to the Research Topic.

Contribution	Research area	Focus	Type of research
1	Wound healing	Network pharmacology and molecular docking analysis	Original article (Jiang et al., 2025)
2	Wound healing	Transcriptome signature	Original article (Shang et al., 2025)
3	Wound healing	Electrospun nanofibers	Original article (Almasi et al., 2025)
4	Wound healing	Electrospun nanofibers	Original article (Mohammadi et al., 2024)
5	UVB damage	Integrated signaling pathways	Original article (He et al.)
6	UVB damage	Homologous recombination repair	Original article (Zhong et al., 2025)
7	Metabolic syndrome with psoriasis	Therapy	Original article (Luo et al., 2025)
8	Generalized pustular psoriasis complicated by acrodermatitis continua of Hallopeau	Therapy	Case report (Zheng et al., 2025)
9	Keratoacanthoma-like lesions	Therapy	Case report (Li et al., 2025)
10	Multiple facial trichoepitheliomas	Therapy	Case report (Gaydina et al., 2024)
11	Alopecia	Inflammation and skin microbiota	Original article (Guan et al., 2025)
12	Alopecia	Mechanism and treatment	Review (Guo et al., 2025)
13	Alopecia	p38/PPAR-γ signaling pathway	Original article (Park et al., 2024)
14	Pruritus	Cannabis and cannabinoids	Review (Sermsaksasithorn et al.)

Skin wounds represent a significant global health Research Topic, as they can be a sign of an underlying disease and require specialized care to heal properly. When a reparative process rather than regeneration occurs, nonfunctional fibrotic tissue masses, namely scars, develop (Marshall et al., 2018). Currently, empirical treatments often rely on a one-size-fits-all approach, failing to consider individual patient characteristics. Thus, they have several limitations, such as 1) a lack of standardization and evidence; 2) ineffectiveness; and 3) practical restrictions. Notably, side effects and complications, i.e., skin atrophy following corticosteroid injection in hypertrophic scars, could be difficult to predict and can delay proper management, leading to suboptimal outcomes, patient dissatisfaction, and increased healthcare costs. In recent years, as reported in *Contribution 1*, network pharmacology and molecular docking (Sakuludomkan et al., 2025) have emerged as powerful tools in drug discovery and development, facilitating the identification of potential molecular targets and elucidation of drug-target interactions. Several advances have deepened our understanding of the molecular mechanisms underlying impaired wound healing (Holl et al., 2021). Unlike normal healing, diabetic wounds exhibit a weak initial inflammatory response followed by 1) a shift toward chronic inflammation; 2) a delayed and inadequate influx of inflammatory cells; 3) low levels of key cytokines like IL-6 and IL-8; and 4) an altered production of enzymes (i.e., neutral endopeptidases) or signaling molecules (i.e., VEGF, FGFs, NF-κB, NLRP3, Wnt/β-catenin and MAPK/ERK) (Patel et al., 2019). To improve the healing process, strategies have been developed to 1) suppress prolonged inflammation and promote the switch of M1 pro-inflammatory macrophages to the alternative, anti-inflammatory M2 phenotype (Cai et al., 2023), or 2) prevent chronic and excessive immune responses by modulating the initial wound phase (Cai et al., 2023; Zhao et al., 2024). Recent advances in technology have begun to enable personalized medicine in dermatology and to generate detailed molecular profiles for diagnosing and monitoring skin conditions (Sherrill et al., 2021). As discussed in *Contribution* 2, identifying transcriptomic characteristics in the early phase of skin wounds could be crucial for developing effective therapeutics (Park et al., 2022) and personalizing wound healing. Individualized medication is essential to achieving optimal patient outcomes at lower cost, but several Research Topic (e.g., genetic underrepresentation, integrating complex data, patient monitoring, and data privacy concerns) remain critical limitations to the development of new therapies. The precision medicine approach provides tools to classify patients with skin injuries into subclasses based on biomarkers that may predict responses to specific therapies. Additionally, this stratification may be used for patients undergoing elective surgery to provide personalized preventive measures and manage the risk of skin scarring. In personalized medicine, 3D biomaterials, such as nanofibers (Kamble et al., 2017), could also be used to create customized wound dressings and tissue-engineered skin grafts for targeted therapies (Cubo-Mateo and Gelinsky, 2021). This approach involves 3D bioprinting structures with specific properties, such as controlled drug release, antimicrobial activity, and enhanced cell growth, to match patients’ individual needs. By precisely layering biomaterials, including those with nanoscale features like nanotubes, researchers can engineer skin treatments that are more effective than traditional methods. As shown in *Contributions 3* and *4*, electrospun nanofibers and therapeutic agents, such as antimicrobials, growth factors, or anti-inflammatory drugs, can accelerate healing and reduce infection, although some limitations have been identified such as 1) weak mechanical strength and rapid degradation (mainly for natural polymers); 2) generation of acidic byproducts (especially with synthetic polymers); 3) high production costs; and 4) difficulties with large-scale manufacturing (Partovi et al., 2024). Given the inherent bioactivity and biocompatibility of natural polymers, as well as the favorable mechanical properties and tunable degradation profiles of synthetic polymers, combinations of these polymers are often used as temporary dressings for epidermal and dermal grafts and wounds. Nanofibers composed of polyvinyl alcohol (PVA)/polyethylene oxide (PEO)/chitosan (CS) are a promising strategy for wound healing due to their ability to create a moist environment, promote cell adhesion and tissue regeneration, and provide antibacterial properties (Alotaibi et al., 2024). PVA is a synthetic polymer used in biomedical applications due to its nontoxicity, hydrophilicity, and biocompatibility (Koosha et al., 2019). Widely used in electrospinning due to its optimal mechanical properties (Kamoun et al., 2021), it is blended with other polymers, such as chitosan (CS) (Alotaibi et al., 2024), to mimic the extracellular matrix and stimulate *in vivo* collagen production (Ribeiro et al., 2021). Another significant factor in skin damage is oxidative stress induced by ultraviolet B (UVB) rays, which are primarily responsible for skin photodamage (Schuch et al., 2017) and genotoxic effects (Cadet et al., 2015) via direct excitation of pyrimidine nucleobases and formation of cyclobutane pyrimidine dimers (CPDs), pyrimidine (6–4) pyrimidone (6-4 PPs). Therefore, protective measures, such as antioxidants, may help mitigate the harmful effects of UVB radiation. As reported in *Contributions 5* and *6*, through the coordinated stimulation of multiple signaling pathways, or the activation of homologous recombination repair (HRR), natural compounds, including anthraquinones (Lin et al., 2022) and derivatives of vitamins (i.e., retinoids) (Zasada and Budzisz, 2019) can enhance skin care efficacy against UVB-induced photodamage (Zeng et al., 2020), promoting 1) ROS scavenging; 2) dermal collagen balance; 3) inhibition of actinic keratosis; and 4) epidermal thickness restoration. Recently, the therapeutic use of monoclonal antibodies (mAbs) has increased significantly in dermatology. While beneficial, mAbs have been demonstrated to cause severe skin reactions, known as adverse dermatological events (ADEs). These reactions include Stevens-Johnson syndrome (SJS), toxic epidermal necrolysis (TEN), erythema multiforme (EM), and fixed drug eruption (FDE) (Kherallah et al., 2025). In Generalized Pustular Psoriasis, the interleukin-36 receptor inhibitor Spesolimab-a humanized monoclonal antibody (Sehgal et al., 2011)- has demonstrated a higher incidence of lesion clearance than placebo, but also causes infections and systemic drug reactions (Bachelez et al., 2021). As reported in *Contribution 8,* when generalized pustular psoriasis coexists with acrodermatitis continua of Hallopeau, targeting the IL-36 proinflammatory cytokine with Spesolimab can result in a rapid and effective response, with minimal side effects. To determine the risks of Spesolimab, longer and larger trials are warranted in patients with pustular psoriasis (Bachelez et al., 2021). Particular attention must also be focused on establishing a comprehensive skin toxicity treatment and management program in patients treated with Sintilimab, a selective anti–PD-1 antibody, which is mainly used to treat cancers (i.e. Hodgkin lymphoma, non-small cell lung cancer, liver cancer, and metastatic colorectal cancer) and known to cause multiple cutaneous keratoacanthoma-like lesions predominantly manifesting as eczema-like maculopapular rashes, lichenoid reactions, vitiligo-like lesions, and flares of psoriasis (Yang et al., 2020). In *Contribution 9*, the authors report a patient case developing numerous eruptive keratoacanthoma-like lesions after the administration of Sintilimab for rectal adenocarcinoma with liver metastasis. Although eruptive keratoacanthoma-like lesions secondary to Sintilimab are exceptionally rarely reported, physicians should be aware of this cutaneous adverse effect as its use becomes more widespread. Skin lesions caused by mutations in the CYLD gene, which encodes a tumor suppressor that induces apoptosis, remain difficult to manage, as illustrated by the case report in *Contribution 10*. The search for safe, effective skincare products has boosted the popularity of natural products derived from plants, minerals, and animals, offering benefits such as moisturising, anti-ageing, anti-inflammatory, and soothing effects. However, with numerous skincare products claiming to contain natural ingredients, it is essential to critically evaluate the scientific evidence supporting their efficacy and benefits. Natural products, such as the *Periplaneta Americana* extract, have been demonstrated through integrated multi-omics analyses (transcriptomics, metabolomics, microbiome) to promote hair regeneration by activating Wnt signaling, suppressing inflammatory pathways, and restoring microbial balance (*Contribution 11*). However, further studies, including targeted inhibition of key signaling molecules, pathway-specific agonist/antagonist-mediated rescue assays, and *in vivo* validation using genetically modified models, are required to delineate the precise mechanisms through which natural products counteract alopecia areata and androgenetic alopecia, reducing inflammation and normalizing the skin’s microbiota. Although they are effective in treating non-scarring alopecia (*Contributions 11, 12, 13),* metabolic syndrome-associated psoriasis (Contribution 7), or pruritus (Contribution 14), the safety profiles and side effects of plant extracts warrant considerable attention, as they may vary and cause uncontrolled effects.
